# Survivin, Survivin-2B, and Survivin-deItaEx3 expression in medulloblastoma: biologic markers of tumour morphology and clinical outcome

**DOI:** 10.1038/sj.bjc.6602317

**Published:** 2005-01-18

**Authors:** J R Fangusaro, Y Jiang, M P Holloway, H Caldas, V Singh, D R Boué, J Hayes, R A Altura

**Affiliations:** 1 Center for Childhood Cancer Research, Columbus Children's Research Institute (CCRI), College of Medicine and Public Health, The Ohio State University, 700 Children's Drive, Columbus, OH 43205, USA; 2 Department of Pediatrics, College of Medicine and Public Health, The Ohio State University, Columbus, OH, USA; 3 Center for Biopathology, Columbus Children's Research Institute, Columbus Children's Hospital and College of Medicine and Public Health, The Ohio State University, Columbus, OH, USA

**Keywords:** apoptosis, medulloblastoma, Survivin, Survivin isoforms

## Abstract

Survivin is an apoptotic inhibitor that is expressed at high levels in a variety of malignancies. Survivin has four known alternative splice forms (Survivin, Survivin-2B, Survivin-deltaEx3, and Survivin-3B), and the recent literature suggests that these splice variants have unique functions and subcellular localisation patterns. We evaluated 19 fresh-frozen paediatric medulloblastomas for the expression of three Survivin isoforms by quantitative PCR. Survivin was most highly expressed when compared with normal cerebellar tissue. We also investigated Survivin protein expression in 40 paraffin-embedded paediatric medulloblastoma tumours by immunohistochemistry. We found a statistically significant association between the percentage of Survivin-positive cells and histologic subtype, with the large-cell-anaplastic variant expressing Survivin at higher levels than the classic subtype. We also found a statistically significant relationship between the percent of Survivin-positive cells in the tumours and clinical outcome, with higher levels of Survivin correlating with a worse prognosis. In summary, our study demonstrates a role for Survivin as a marker of tumour morphology and clinical outcome in medulloblastoma. Survivin may be a promising future prognostic tool and potential biologic target in this malignancy.

Medulloblastoma is the most common primary malignant central nervous system (CNS) tumour of childhood. It is an invasive embryonal tumour originating in the cerebellum with an inherent tendency to metastasise within the CNS via the cerebrospinal fluid (CSF) (Stavrou *et al*, 2001; Eberhart *et al*, 2003; Mazzola and Pollack, 2003). There are four major histologic subtypes of medulloblastoma including classic, desmoplastic, extremely nodular, and large-cell-anaplastic. Each subtype has unique histologic features and prognosis (Rickert and Paulus, 2001; Kleihues *et al*, 2002). The large-cell-anaplastic variant is clinically the most aggressive subtype, and represents between 4 and 25% of all medulloblastomas cases (Leonard *et al*, 2001; Eberhart *et al*, 2002).

There have been many advances in the treatment of medulloblastoma including improved surgical resection techniques, radiation, and chemotherapy (Zeltzer *et al*, 1999; Packer *et al*, 2003a; Taylor *et al*, 2003). These changes have greatly improved overall survival with current treatment strategies achieving up to 70–75% 5-year disease-free survival (DFS) (Evans *et al*, 1990; Packer *et al*, 1994; Leonard *et al*, 2001). As a consequence of current treatment strategies, however, patients often suffer from life-long disabilities and cognitive dysfunction. It is estimated that 90% of medulloblastoma survivors have impaired intelligence, and 40–100% of long-term survivors have some degree of cognitive dysfunction (Walter *et al*, 1999; Palmer *et al*, 2001; Packer *et al*, 2003b). Radiation and chemotherapy also often lead to significant endocrine abnormalities, including thyroid, growth, and gonadal dysfunction (Xu *et al*, 2004). High morbidity and continued mortality have prompted the search for new treatment strategies as well as for biologic markers that might be used for targeted therapy to minimise current treatment-related side effects. Survivin is an attractive target gene to evaluate for these purposes, as it has been shown to be a significant marker of tumour aggression in a number of malignancies (Altieri, 2003b).

The protein encoded by the Survivin gene has significant structural homology to a group of proteins known as inhibitors of apoptosis (IAP) (Altieri and Marchisio, 1999; Altieri, 2003a). Survivin is expressed at high levels in numerous adult malignancies including lymphomas, breast, lung, and colon carcinoma, as well as in paediatric cancers including neuroblastoma, Wilm's tumour, choroid plexus tumours, and ependymomas (Altieri, 2003b; Altura *et al*, 2003). It is expressed widely during normal embryonic development, but only in a small subset of adult normal differentiated tissues, including the colonic epithelium, uterine endometrium, vascular endothelium, and the subventricular region of normal brain (Konno *et al*, 2000; Gianani *et al*, 2001; Altieri, 2003b; Altura *et al*, 2003). Survivin is expressed in a cell-cycle-dependent manner with the highest levels of expression in G2/M (Skoufias *et al*, 2000). Its predominance in certain normal and malignant tissues suggests that it has a specific function within them. Previous reports have suggested that the function of Survivin in cancer is predominantly as an IAP, blocking mitochondrial-dependent apoptosis (Li *et al*, 1998; Altieri and Marchisio, 1999). Survivin is also involved in a mitotic checkpoint as a chromosomal passenger protein (CPP). This family of proteins aligns the chromosomes appropriately during mitosis and maintains accurate cell division in normal cells (Lens *et al*, 2003). It also prevents the development of abnormal numbers of chromosomes that may occur during the transition from a nonmalignant to a malignant phenotype.

The human Survivin gene locus has four known splice variants that result from alternative splicing of its messenger RNA. It has been hypothesised that each of these isoforms has a unique function and a unique subcellular localisation pattern (Mahotka *et al*, 1999, 2002a, 2002b). Survivin and the Survivin isoforms have been determined to be markers for tumour aggression and clinical outcome in CNS tumours (Sasaki *et al*, 2002; Katoh *et al*, 2003; Yamada *et al*, 2003). These studies focused primarily on adult CNS tumours, and often, tumours of two or more differing morphologies. Survivin expression has not been previously evaluated in a series of medulloblastoma tumours. In the current work, we evaluate the expression of Survivin and its isoforms as potential biologic and prognostic markers in medulloblastoma.

## MATERIALS AND METHODS

### Patient recruitment

Tumour samples obtained from 40 paediatric patients with a diagnosis of medulloblastoma were selected from the Columbus Children's Hospital (CCH) pathology database based on diagnosis and availability of paraffin-embedded tumour material. All patients were treated at CCH between 1992 and 2003. Studies were performed following approval from the CCH Internal Review Board (IRB). Treatment was based on diagnosis and extent of surgical resection. Patients received a combination of surgery, radiation and/or chemotherapy. Additionally, 19 fresh-frozen tumours were obtained from the Cooperative Human tumour Network (CHTN), after approval through the CCH IRB.

### Immunohistochemistry

Paraffin-embedded tumour samples were sectioned at a thickness of 4–5 *μ*m on a Leica microtome and placed on charged slides. Heat antigen retrieval was performed on a pressure cooker with Antigen Retrieval Citra (BioGenex, San Ramon, CA, USA) according to the manufacturer's instructions. Blocking was performed with Powerblock for 10–15 min. The samples were incubated at 4°C overnight with either the anti-Survivin primary antibody NB-500-201 (Novus Biologicals, Littleton, CO, USA) or the sc-10811 antibody (Santa Cruz Biotech, CA, USA). Immunohistochemistry was performed using the avidin–biotin method with reagents from Biocare Medical (Walnut Creek, CA, USA). Colour development was achieved by applying peroxidase substrate chromagen AEC (Dako, Carpentenia, CA, USA). Slides were counterstained with Mayer's haematoxylin and mounted in aqueous mounting solution (Crystal Mount, Biomeda, Foster City, CA, USA).

Each slide was evaluated for Survivin expression by two investigators, independently. A total of 500 cells were counted from an overall representative and well-preserved region of the slide. A repeat cell count was performed by the neuro-pathologist on samples in which there was greater than a 3% difference between the two investigators.

### Western blotting

Daoy cells (ATCC #HTB-186) are human medulloblastoma cells originally established from a medulloblastoma tumour isolated from the posterior fossa of a 4 year-old child (Jacobsen *et al*, 1985). Cells were grown in Dulbecco's modified essential medium supplemented with 10% fetal bovine serum and 2 mM L-glutamine. Protein was extracted from Daoy cells using RIPA lysis buffer. The SDS boiling method was used to isolate protein from fresh-frozen tissue. Samples were homogenised with a mortar and pestle in a heated buffer solution (1% SDS and 10 mMTris, pH 7.4). Equal amounts of protein (150 *μ*g) were separated on an 18% SDS–PAGE and transferred onto a nitrocellulose membrane using a semidry transfer apparatus. The membrane was probed with the anti-Survivin polyclonal antibodies (NB-500-201 and sc-10811) (1 : 5000) and HRP-conjugated anti-rabbit IgG (1 : 5000). Detection was performed using the advanced ECL System (Amersham, Piscataway, NJ, USA).

### RNA isolation

Total RNA was isolated from fresh-frozen brain tumour tissue using RNeasy Mini Kit. Briefly, the tumour sample was weighed, then placed in a 5 × lysis buffer containing guanidine isothicyanate. The mixture was homogenised and purified on an RNeasy mini column (Valencia, CA, USA).

### Real-time PCR

Omniscript Reverse Transcriptase (Qiagen, Hilden, Germany) was used to synthesise cDNA from total RNA. Real-time PCR was performed using an ABI 7700 (Advanced Bio-systems, Foster City, CA, USA). The sequences used for primers and probes for Survivin-2B included the following: forward 5′-GCACGGTGGCTTACGCCTG, probe 5′-FAM-ATACCAGCACTTTGGGAGG, and reverse 5′-ACCGGACGAATGCTTTTTATGTTCC. The sequences for Survivin-deltaEx3 were forward 5″-GCTGGGAGCCAGATGACG, probe 5′ FAM-CCCCATGCAAAGGAAACCAACAATAAGAA and reverse 5′-TTCGCAGTTTCCTCAAATTCTTT. Real-time probe sets were selected using the Primer Express program (Applied Biosystems, Foster City, CA, USA). Probes were labelled with FAM dye and TAMRA quencher. Each PCR reaction was performed in triplicate. Ribosomal RNA was used as an internal control for each sample. Standard curves were constructed using serial dilutions of RNA isolates from a medulloblastoma cell line (Daoy). The results from each sample were compared with normal cerebellum as a calibrator using the Relative Standard Curve method (Applied Biosystems, Foster City, CA, USA).

### Statistical methods

Data were illustrated with box plots and Kaplan–Meier curves. Percent staining was compared across subtypes with an overall nonparametric Kruskal–Wallis test and followed up with Wilcox on rank sums paired comparisons. The association between per cent staining and survival was evaluated with a Cox proportional hazards survival test. Differences were considered statistically significant at an alpha of 0.05.

## RESULTS

### Expression of Survivin isoforms in medulloblastoma

**Figure 1:**
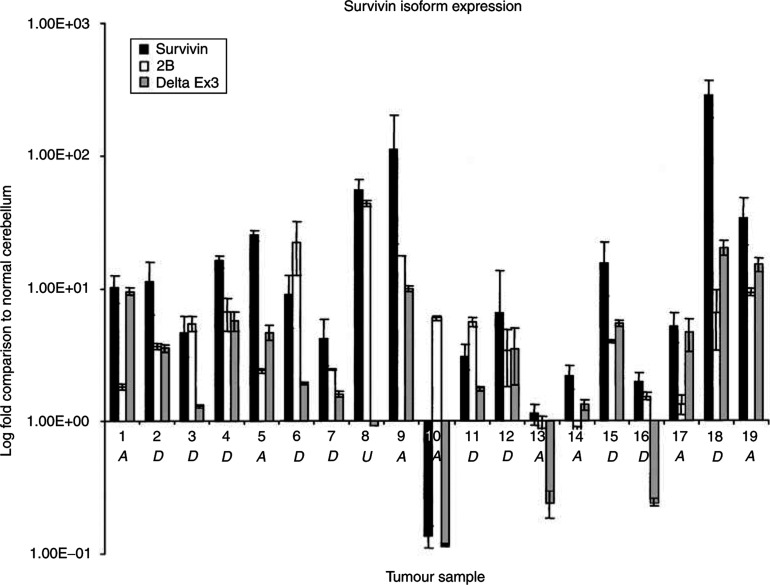
Transcriptional expression of Survivin, Survivin-2B, and Survivin-deltaEx3 in 19 fresh-frozen medulloblastoma tumours using quantitative PCR. Fold expression compared to normal human cerebellum was calculated using the Relative Standard Curve Method (ABI). ‘A’=Alive, ‘D’=Dead, and ‘U’=Unknown outcome status.

We evaluated the transcriptional expression of three of the different Survivin isoforms by quantitative PCR in 19 different fresh-frozen medulloblastoma samples. The expression of Survivin, Survivin-2B, and Survivin-deltaEx3 in the tumour samples was quantified using the Relative Standard Curve Method and then compared with the expression in normal cerebellum. The results indicate that the isoforms are expressed in the majority of tumour samples at levels that are higher than their expression in normal cerebellum. The relative expression of Survivin, when compared to Survivin-2B and Survivin-deltaEx3 was the highest in the majority of tumour samples. Expression levels of Survivin in the tumours ranged from <1 (one sample) to 278-fold above normal human cerebellum (Figure 1). Survivin-2B levels ranged from <1 (2 samples) to 43-fold above normal human cerebellum, and levels of Survivin-deltaEx3 ranged from <1 (4 samples) to 20-fold above normal cerebellum (Figure 1).

**Figure 2:**
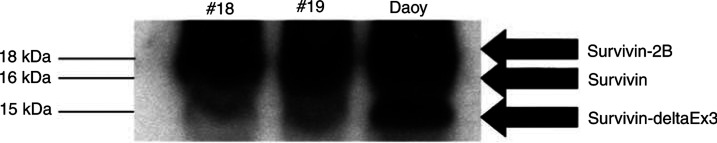
Protein expression in Daoy cells and medulloblastoma tumours. Daoy cell line and two medulloblastoma tumour samples (#18 and #19) stained with the anti-Survivin antibody Novus-500-201 demonstrated three bands at the predicted molecular weights of Survivin, Survivin-2B, and Survivin-deltaEx3.

To evaluate whether these isoforms were translated into stable protein in medulloblastoma cells, Western blots were performed on a subset of these tumour samples and on lysates made from Daoy cells using the NB-500-201 polyclonal antibody (Figure 2). The Western blots showed specific bands at the predicted molecular weights for Survivin and the Survivin isoforms, Survivin-2B and Survivin-deltaEx3, suggesting that these transcripts are translated in these tumour cells. The same results were obtained using the sc-10811 antibody (results not shown).

### Patient outcome in 40 patients with medulloblastoma

**Figure 3:**
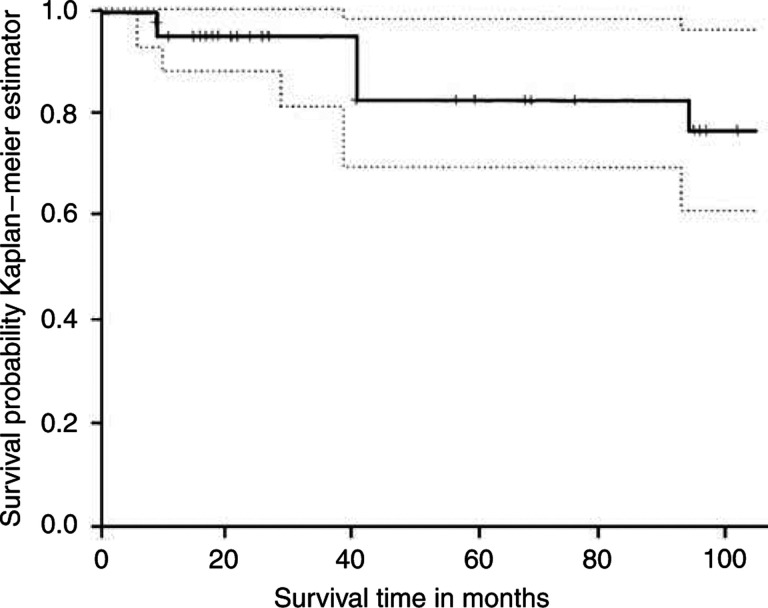
Kaplan–Meier curve of overall survival (OS) in 40 medulloblastoma patients, from our study population. The solid line represents OS, and the dotted lines represent the 95% confidence intervals.

Among the 40 patients with medulloblastoma, the mean and the median ages were 8.4 and 8 years old, respectively. The male : female ratio was 1.85 : 1. The medulloblastoma tumours were classified into the following subtypes: four large-cell-anaplastic (10%), four desmoplastic (10%), one extremely nodular (2.5%), and 31 classic (77.5%). The median follow-up time on living patients was 57 months. The most recent follow-up showed that 77.5% of the original 40 patients were still alive, and the 5-year overall survival (OS) was approximately 82% (Figure 3).

### Survivin expression in medulloblastoma correlates with morphologic subtype and clinical outcome

**Figure 4:**
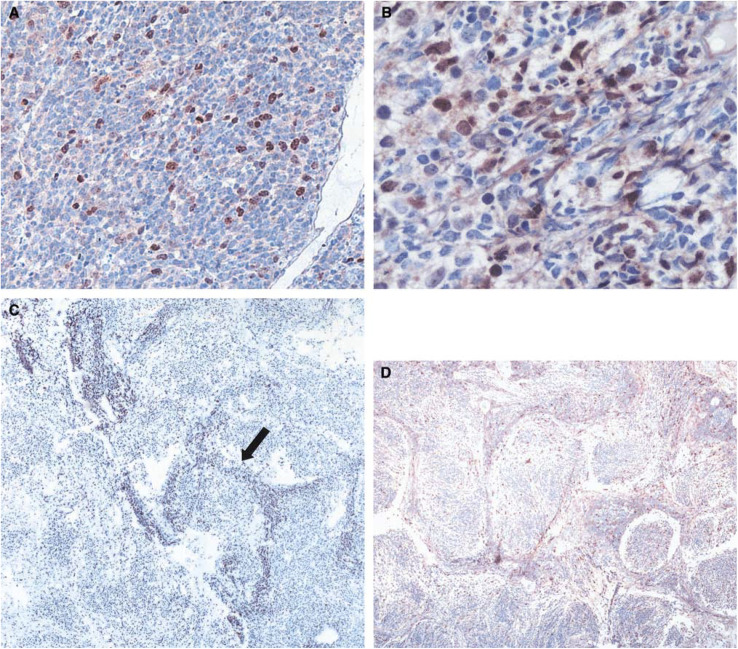
Protein expression of Survivin in medulloblastoma tumours. Immunohistochemistry of (**A**) classic (× 140), (**B**) large-cell-anaplastic (× 270), (**C**) desmoplastic (× 70), and (**D**) extremely nodular medulloblastoma (× 70) using NB-500-201 antibody. Red staining indicates Survivin-positive cells. Note the specific nuclear localisation in all of the Survivin-positive cells. The arrow in (**C**) indicates the high percentage of staining in the internodular areas of a desmoplastic variant.

Evaluation of the 40 medulloblastoma tumours by immunohistochemical staining (both NB-500-201 and sc-10811) showed that Survivin localised to the nucleus in all tumour cells that expressed Survivin (Figure 4). The percentage of positive cells ranged from <5% to a maximum of 40%. The large-cell-anaplastic subtype showed a higher overall percentage of positive cells (Figure 4B), whereas the desmoplastic and extremely nodular subtypes showed a unique pattern of Survivin staining (Figures 4C and D). The internodular (poorly differentiated) areas had higher percentages of Survivin staining when compared with the intranodular (highly differentiated) regions.

**Figure 5:**
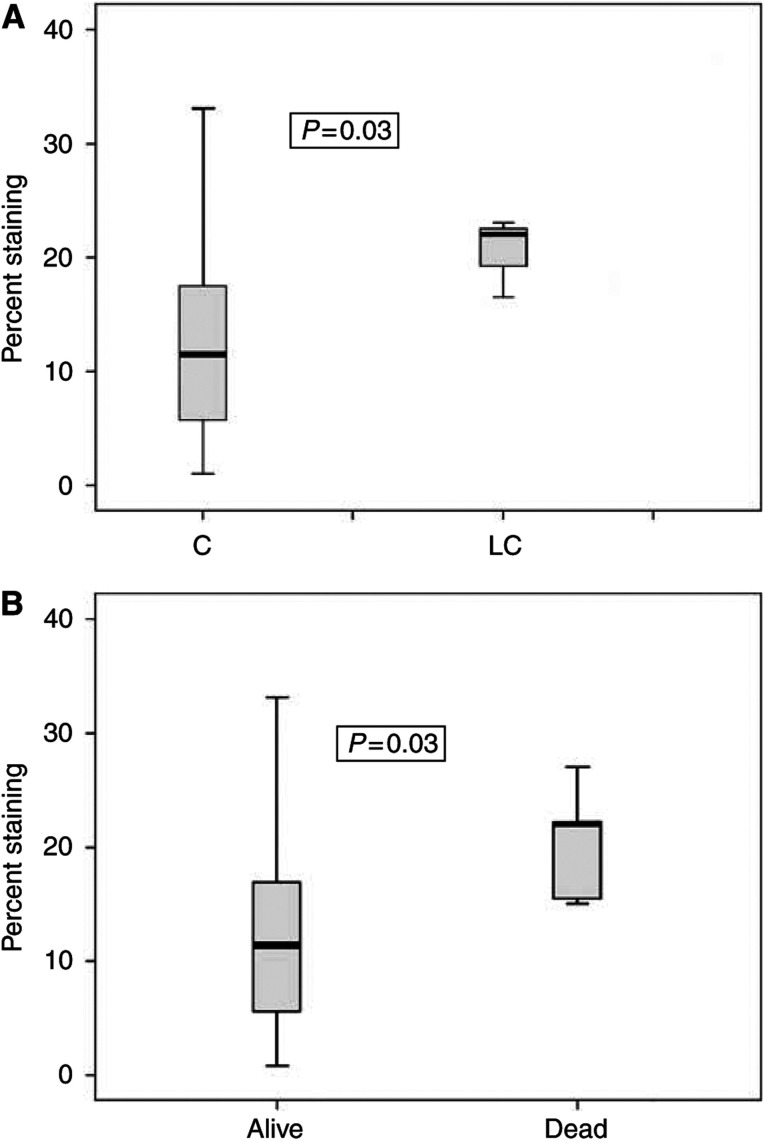
Box plot graphical representation of percent of cells staining positive with the NB-500-201 antibody in large-cell-anaplastic (LC) tumours *vs* classic tumours (C) (**A**), and in tumours of deceased patients *vs* those in living patients (**B**). The thick horizontal line within the box represents the mean value. The box itself represents the 25–75% range of data, and the vertical bars extending from the box represent the extreme values.

The percentage of Survivin-positive cells within each sample did not correlate with age or sex of the patient. A correlation between morphologic subtype and percent Survivin staining was statistically significant with the NB-500-201 antibody (Figure 5A). As the percentage of cells staining positive for Survivin increased, the tumour was statistically more likely to be a large-cell-anaplastic variant. A paired comparison between the classic undifferentiated and the large cell variant revealed a statistically significant difference (*P*=0.03). The mean percentage of staining was 8–10% higher in the large-cell-anaplastic subtype *vs* the classic subtype of medulloblastoma. These results suggest that Survivin may be a marker for tumour aggression and useful as a supplemental tool distinguishing the large-cell-anaplastic variant from the classic medulloblastoma subtype.

The percentage of cells staining positive for Survivin also correlated with clinical outcome. Tumours of deceased patients had a higher mean percentage of Survivin-positive cells when compared with living patients (Figure 5B) (*P*=0.03). This supports a role for Survivin as a marker for clinical outcome in medulloblastoma. When the length of time from diagnosis to death or diagnosis to most recent follow-up was taken into consideration, the relationship between percent staining and survival approached, but lost, true statistical significance (*P*=0.1). This may indicate that a larger study population is necessary to appreciate a statistically significant relationship for this parameter.

## DISCUSSION

Proteins from the IAP family play an important role in inhibiting pathways leading to programmed cell death (Li *et al*, 1998; Altieri and Marchisio, 1999; Altieri, 2003a). Although the functional roles of Survivin and the Survivin isoforms in normal cells *vs* their roles in tumourigenesis are not yet completely understood, transcriptional and protein levels of Survivin and its isoforms have been determined to correlate with tumour aggression in a variety of malignancies, including CNS tumours (Mahotka *et al*, 2002b; Altieri, 2003b; Altura *et al*, 2003, Kleinschmidt-DeMasters *et al*, 2003; Shariat *et al*, 2004). A study evaluating Survivin expression in adult gliomas showed that patients with high levels of Survivin protein expression had significantly worse clinical outcomes. The prognostic value of Survivin expression was independent of histology alone in this study (Chakravarti *et al*, 2002). Another group evaluated the transcriptional expression of the Survivin isoforms by quantitative PCR in primary CNS tumours. These authors concluded that Survivin was preferentially expressed in malignant brain tumours and gliomas when compared with benign and nonglial tumours, respectively. They also stated that higher expression levels of Survivin-deltaEx3 were detected in malignant tumours, while Survivin-2B was more prominent in benign tumours (Yamada *et al*, 2003). This is one of the first suggestions that each Survivin isoform might have a unique function in CNS tumours.

In our study, Survivin and its isoforms were expressed at much higher levels in the medulloblastoma tumours when compared with normal human cerebellum. This finding suggests that the Survivin gene plays an active role in tumourigenesis in medulloblastoma. Quantification of the expression levels of the alternative splice forms of Survivin in medulloblastoma compared to normal cerebellar expression showed a predominance of Survivin compared to Survivin-2B and Survivin-deltaEx3 in the 19 medulloblastoma samples in our study. Survivin-2B was the next highest expressed isoform, and Survivin-deltaEx3 was the lowest expressed, albeit still at higher levels (up to 20-fold) that of normal cerebellum. Prior functional studies using exogenous expression constructs of Survivin-2B and Survivin-deltaEx3 in different cell lines suggested that Survivin-deltaEx3 protects cells from programmed cell death, while Survivin-2B enhances programmed cell death (Islam *et al*, 2000; Mahotka *et al*, 2002a, 2002b).

Given the highly malignant nature of medulloblastoma, we hypothesise that the dominant Survivin isoform expressed in these tumours has a critical antiapoptotic function. Our data showing that Survivin, itself, is the highest expressed transcript of all the isoforms in medulloblastoma tumours is consistent with this hypothesis. We also hypothesise that Survivin-deltaEx3 likely functions as an antiapoptotic protein in the formation and progression of medulloblastoma. As seen in previous studies, the ratio of Survivin isoforms to each other may be critical for their functions (Yamada *et al*, 2003). It will be interesting to quantify the protein products of each of the Survivin isoforms in medulloblastoma once sensitive and specific ELISA assays become available for Survivin-2B and Survivin-deltaEx3. Targeting the isoform protein(s) that are most functionally active or tumourigenic in a specific tumour may be the most effective anti-Survivin treatment strategy.

Interestingly, our immunohistochemical staining of primary medulloblastoma tumours showed a unique nuclear pattern of expression with each of two different polyclonal anti-Survivin antibodies tested. Previously published results by our laboratory, evaluating Survivin expression in normal human brain cells, showed that Survivin localised to the nucleus with one antibody, but was predominantly cytoplasmic with the other antibody (Altura *et al*, 2003). Immunofluorescent studies performed in HeLa cells using antibodies directed against specific amino acids within the Survivin protein have shown that Survivin localises to different subcellular nuclear and cytosolic structures (Fortugno *et al*, 2002). In that study, the nuclear Survivin pool was associated with kinetochores and was hypothesised to be a potentially important regulator of the mitotic spindle checkpoint. The specific nuclear staining pattern found in our medulloblastoma cases suggests that the function of the Survivin proteins in medulloblastoma is related to their nuclear localisation, or perhaps, mislocation to the nucleus.

Among the different morphologic subtypes, the desmoplastic and extremely nodular medulloblastomas in our study population had unique Survivin staining patterns with an increased number of Survivin-positive cells in the internodular zones in areas of poor neuronal differentiation, as indicated by the arrow in Figure 4C. Historically, the desmoplastic and extremely nodular subtypes have improved survival rates, possibly because they are more differentiated, slower growing, and more sensitive to chemotherapy and radiation (Packer *et al*, 1999; Suresh *et al*, 2003). The high percentage of internodular Survivin staining in our samples suggests that Survivin may play an important role in tumourigenesis in these tumour subtypes as well.

The large-cell-anaplastic tumours in our study population had a statistically higher mean percentage of Survivin-positive cells when compared with the classic medulloblastoma tumours. Histopathologically, the large-cell-anaplastic variant is composed of large neoplastic cells with large, round, vesicular nuclei, and prominent nucleoli. There are frequent numbers of mitotic cells, abundant number of cells undergoing apoptosis, with increased areas of anaplasia (Brown *et al*, 2000; Leonard *et al*, 2001). A Pediatric Oncology Group (POG) study evaluating medulloblastomas showed that the large-cell-anaplastic subtype was more likely to present with malignant cells within the CSF and distant metastasis compared to non-large-cell tumours. The overall survival of these patients was markedly inferior to non-large-cell medulloblastoma patients (Brown *et al*, 2000). Overall, clinical outcomes for patients with large-cell-anaplastic medulloblastoma are poor, with about a 20% 5-year OS (McManamy *et al*, 2003). Although we can subclassify these tumours by histology, thus far, current therapy has not been successful in eradicating these tumours. Our study suggests that immunohistochemical staining with Survivin antibodies may be a useful tool to identify the more aggressive large-cell-anaplastic subtypes. Survivin may be a potential biologic target in this particular subgroup of patients.

Advances in preclinical anti-Survivin therapies have been evaluated in numerous tumours and tumour cell lines. Antisense oligonucleotides to Survivin have shown potential therapeutic responses in mesothelioma, breast, and lung cancer (Tamm *et al*, 1998; Olie *et al*, 2000; Xia *et al*, 2002). Immune-modulated therapies have also been evaluated. In a phase I study, an HLA-A24-restricted antigenic peptide, Survivin 2B80-88, which is recognised by CD8+ cytotoxic T-lymphocytes, was used to vaccinate patients with recurrent colorectal cancer. They observed a decrease in tumour markers in 40% of patients tested and a reduction in tumour size in one of 15 patients (Tsuruma *et al*, 2004). These and future Survivin-targeted therapies may be one element of biologic therapy that would also benefit children with medulloblastoma.

Our results show that the Survivin proteins are biologic markers of tumour morphology and clinical outcome in medulloblastoma patients, suggesting they may be useful molecular targets in these tumours. Specific biologic aberrations in medulloblastoma will become increasingly more important therapeutically as an attempt is made to decrease the high morbidity associated with current medulloblastoma treatment strategies. Early studies evaluating anti-Survivin therapies in other malignancies and tumour cell lines have shown promising results (Xia *et al*, 2002; Tsuruma *et al*, 2004). The use of novel biologic therapies in combination with conventional chemotherapy and radiation will be the future key to decreasing mortality in high-risk patients and reducing morbidity in all medulloblastoma patients. Our findings suggest that future large clinical studies of medulloblastoma should evaluate Survivin and its isoforms as markers of tumour morphology, predictors of patient outcome, and targets for future biologic therapies.
